# High Smac/DIABLO expression is associated with early local recurrence of cervical cancer

**DOI:** 10.1186/1471-2407-6-256

**Published:** 2006-10-26

**Authors:** Abril Arellano-Llamas, Francisco J Garcia, Delia Perez, David Cantu, Magali Espinosa, Jaime G De la Garza, Vilma Maldonado, Jorge Melendez-Zajgla

**Affiliations:** 1 Subdirección de Investigación Básica, Instituto Nacional de Cancerología, Av. San Fernando # 22, Tlalpan 14080 México City, Mexico; 2 Departamento de Anatomia Patologica, Instituto Nacional de Pediatria, Insurgentes Sur # 3700, Coyoacan 04530 México City, Mexico; 3 Subdireccion de Patologia, Instituto Nacional de Cancerología, Av. San Fernando # 22. Tlalpan 14080 México City. Mexico; 4 Subdirección de Cirugía, Instituto Nacional de Cancerología Av. San Fernando # 22. Tlalpan 14080 México City. Mexico

## Abstract

**Background:**

In a recent pilot report, we showed that Smac/DIABLO mRNA is expressed *de novo *in a subset of cervical cancer patients. We have now expanded this study and analyzed Smac/DIABLO expression in the primary lesions in 109 cervical cancer patients.

**Methods:**

We used immunohistochemistry of formalin-fixed, paraffin-embedded tissue sections to analyze Smac/DIABLO expression in the 109 primary lesions. Seventy-eight samples corresponded to epidermoid cervical cancer and 31 to cervical adenocarcinoma. The median follow up was 46.86 months (range 10–186).

**Results:**

Smac/DIABLO was expressed in more adenocarcinoma samples than squamous tumours (71% vs 50%; p = 0.037). Among the pathological variables, a positive correlation was found between Smac/DIABLO immunoreactivity and microvascular density, a marker for angiogenesis (p = 0.04). Most importantly, Smac/DIABLO immunoreactivity was associated with a higher rate of local recurrence in squamous cell carcinoma (p = 0.002, log rank test). No association was found between Smac/DIABLO and survival rates.

**Conclusion:**

Smac/DIABLO expression is a potential marker for local recurrence in cervical squamous cell carcinoma patients.

## Background

Apoptosis is a type of programmed cell death morphologically characterized by membrane blebbing, nuclear and cytoplasmic condensation and the formation of vesicles containing intact organelles [1]. Deregulation of this process has been implicated in numerous pathological conditions including cancer. Two well-characterized apoptotic pathways converge in caspase activation: the death receptor pathway and the intrinsic or mitochondrial pathway [2]. The intrinsic pathway is triggered by various extracellular and intracellular stresses such as growth-factor withdrawal, hypoxia, DNA damage and oncogene induction. These signals converge on the mitochondria, resulting in the regulated release of cytochrome c and other proapoptotic molecules. This leads to assembly of the apoptosome, a complex containing cytochrome *c*, apoptotic protease activating factor 1 and caspase 9. This protein complex in turn initiates the activation of effector caspases [3]. To avoid unregulated activation of these enzymes, members of the Inhibitors of Apoptosis Proteins (IAPs) family bind caspases and prevent their activation, thus tightly controlling the induction of apoptosis [4]. In cells undergoing apoptosis, IAPs are inactivated by interacting with proteins containing the so-called IBM (IAP-binding motif) [4,5]. One of these IBM proteins is the recently identified Smac/DIABLO. Smac is a proapoptotic protein that in healthy cells resides in the intermembrane space in the mitochondria [6,7] but is released into the cytosol during apoptosis, where it interacts with IAPs and disrupts their ability to bind caspases [8]. Smac interacts with all mammalian IAPs examined so far: XIAP, cIAP-1, cIAP-2, survivin and ML-IAP [6,7,9,10]. In addition to its ability to disrupt caspase inhibition by IAPs, Smac delivers cIAP1 and cIAP2 for rapid degradation by autoubiquitination [11]. The balance of Smac/DIABLO and IAPs determines the threshold for a variety of apoptotic stimuli.

IAPs are commonly over-expressed in human cancers [12-14], contributing to the carcinogenic process and to the resistance of these cells to chemotherapy [15]. However, the importance of Smac/DIABLO, a partner in the caspase inhibitory system, has been less well explored. Patients with lower Smac/DIABLO levels in lung [16] and renal [17] tumors have been shown to have worse prognoses. This indicates that Smac expression may play a role in the progression of several neoplastic diseases and could be useful for prognosis. In a recent pilot report using RT-PCR, we showed that Smac/DIABLO is expressed *de novo *in a subset of cervical cancer patients [18]. The study only analyzed 41 cancer samples, precluding a more detailed analysis. In the present work we have analyzed Smac/DIABLO expression in formalin-fixed, paraffin-embedded tissue sections from primary lesions in 109 cervical cancer patients.

## Methods

### Tumor samples

One hundred and nine cervical cancer samples were obtained from consecutive patients recruited in the Gynecological Cancer Clinic at the Instituto Nacional de Cancerologia of Mexico between January 1990 and December 1993. Seventy-eight corresponded to epidermoid cervical cancer and 31 to cervical adenocarcinoma. Written consent was obtained from the patients before the samples were collected. Tumors were staged according to the International Gynecology and Obstetric Federation (FIGO) system. The samples comprised 6 in stage IB, 2 in IIA, 62 in IIB, 15 in IIIA and 24 in IIIB.

### Histology

Histopathology was graded according to the WHO (World Health Organization) classification system.

### Immunohistochemistry

To perform immunohistochemistry, 4 μm thick sections were obtained from archival blocks, deparaffinized and rehydrated in graded ethanol. Antigen retrieval was done by heating the slides (5 min in a microwave oven) in citrate buffer at pH 6.0. Endogenous peroxidase activity was blocked with 3% H_2_O_2 _for 5 min. Sections were incubated with primary antibody at 1:75 dilution (Anti-Smac/DIABLO, Calbiochem Cat: PC574) for 30 min at room temperature, rinsed with PBS, and reincubated with a secondary antibody (Rabbit Polydetector Bio SB. Cat BSB0221) for 30 min. The peroxidase substrate was diaminobenzidine (DAKO, Denmark). Slides were finally counterstained with Gill's hematoxilin for 8 min. As expected from our previous results [18], Smac/DIABLO staining was undetectable in normal cervix (not shown). To further validate the staining, three aleatory biopsies of gastric carcinoma were used as positive controls, based in a previous report showing that 70% of these tumors presented positive staining [19]. All three biopsies showed a strong positive cytoplasmic signal. Negative controls were included to verify specificity. The antibody used reacts specifically with Smac/DIABLO, as verified by western blot assays of cervical cancer cells with depleted (using an antisense approach) or overexpressed Smac (Ceballos, G., submitted)

### Microvessel Density (MVD)

Microvessel density was determined as described in [20]. Briefly, immunostaining for CD34 was performed in using a monoclonal antibody (anti CD34 monoclonal Class II, clone QBEnd/10, DAKO, Denmark) with a commercial kit, as directed by the manufacturer (LSAB peroxidase, DAKO, Denmark). Areas of cancer tissue with distinct microvessels ("hot spots") were selected and microvessel density was determined by counting all vessels at a total magnification of 200×. All slides were evaluated blindly and independently by two pathologists. Routine haematoxylin and eosin was used to evaluate grade and lymphovascular involvement.

### Evaluation of Smac/DIABLO expression

Smac/DIABLO immunoreactivity was evaluated by two observers in a blind procedure. The slides were assessed for intensity (0: negative, 1: less intense than positive control, 2: equal intensity to control, 3: more intense than control) and positive area (percentage).

### Statistical analysis

To investigate correlations between clinical variables and Smac/DIABLO positive immunostaining we used *Chi *square tests (stage, histology and grade) and Mann-Whitney tests (age, lymphovascular permeation and microvascular density). Survival and recurrence were evaluated using Kaplan-Meier curves for each variable and the curves were compared by a log rank test. The statistical package SPSS 12.0 was used for analyses and statistical significance was accepted when the *p *value was less than 0.05.

## Results

The present study included 109 patients with a mean age of 47.14 years (range 25–73), similar to reports elsewhere [20]. Thirty-one of them corresponded to adenocarcinoma and 78 to squamous cell carcinoma. The median age at diagnosis was 47 years, and the median follow up was 46.86 months (range 10–186). The patients were mostly in advanced stages, distributed as follows: 6 cases corresponded to stage I, 64 to stage II and 39 to stage III. Histologically, 11% of the samples were well differentiated, 33% moderately well differentiated and 56% poorly differentiated.

**Figure 1:**
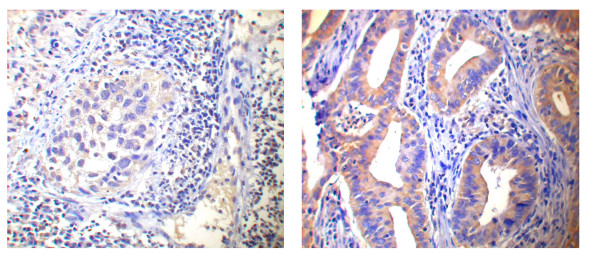
Photomicrographs showing Smac/DIABLO immunoreactivity in representative cervical cancer samples. Staining was predominantly cytoplasmic and involved only tumor cells. Left panel: low intensity (1) score. Right panel: high intensity (2) score. Total magnification: 400×. Procedures were performed as described in "Materials and Methods".

Sixty-one (56%) of the patients presented positive immunostaining for Smac/DIABLO, a slightly higher percentage that in our previous report (31.7%) [18]. Staining was predominantly cytoplasmic and involved only tumor cells; stromal cells were negative (Fig. 1). A similar number of samples from different stages or histopathological grades were positive for Smac/DIABLO (Table 1). Interestingly, a larger number of adenocarcinoma samples were Smac immunopositive (71% vs 50%; p = 0.037) (Table 1). Among the pathological variables, we found that positive Smac/DIABLO samples presented a higher microvascular density, a factor previously shown to have prognostic significance in cervical cancer (p = 0.04) [20] (Table 2). No other correlation was found (not shown).

**Table 1:** Smac/DIABLO positive immunoreactivity in cervical cancer samples.

Variable	**No. patients **(n = 109)*	*p*
**FIGO stage**		
I	5 (83)	0.403
II	35 (54)	
III	21 (53)	
		
**Histology of tumors**		
Squamous cell	39 (50)	0.037
Adenocarcinoma	22 (71)	
		
**Grade**		
Well differentiated	9 (75)	0.19
Moderately differentiated	22 (61)	
Poorly differentiated	30 (49)	
		
**Current status**		
Alive	32(59)	0.121
Dead	29(53)	

*Number of patients expressing Smac/DIABLO and percentage of positive patients in the group (in parenthesis). Chi square was used to test for differences. No significant differences were found when analyses were performed in data dichotomized for histology.

**Table 2:** Smac/DIABLO positive immunoreactivity in squamous cervical cancer samples.

Smac/DIABLO	Median	*AD*	*p*
	**Age**		
Negative	46.58	9.17	0.621
Positive	47.52	10.6	
			
	**LVP**		
Negative	0.51	0.72	0.479
Positive	0.39	0.63	
			
	**MVD**		
Negative	18.02	9.7	0.047
Positive	22.93	8.93	

Age, Lymphovascular Permeation (**LVP**) and Microvascular Density (**MVD**) values for positive and negative Smac/DIABLO patients. ***AD***: Absolute Deviation. Mann-Whitney test was used to verify significant differences. Similar differences were found when analyses were performed in adenocarcinoma samples.

**Figure 2:**
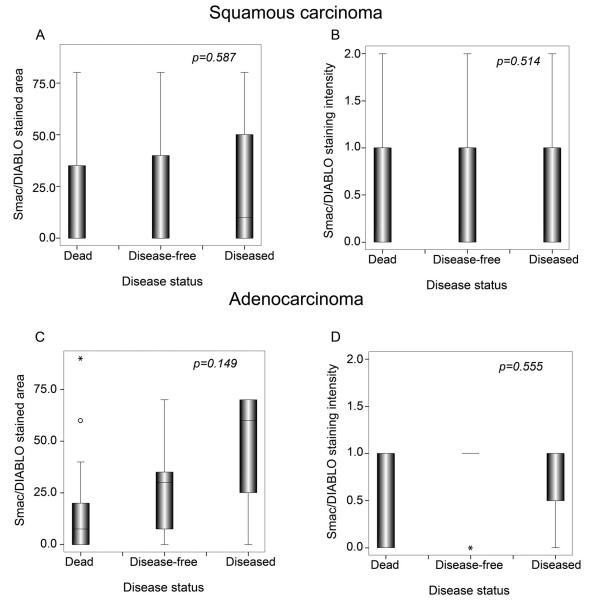
Smac/DIABLO immunostaining does not correlate with disease status. (A) and (C): Smac/DIABLO immunostained area. (B) and (D): Smac/DIABLO intensity of immunostaining. Scores were calculated as described in Material and Methods. Graphs show median, upper and lower quartiles. P value testing the significance of the difference was determined by *Chi *square test.

**Figure 3:**
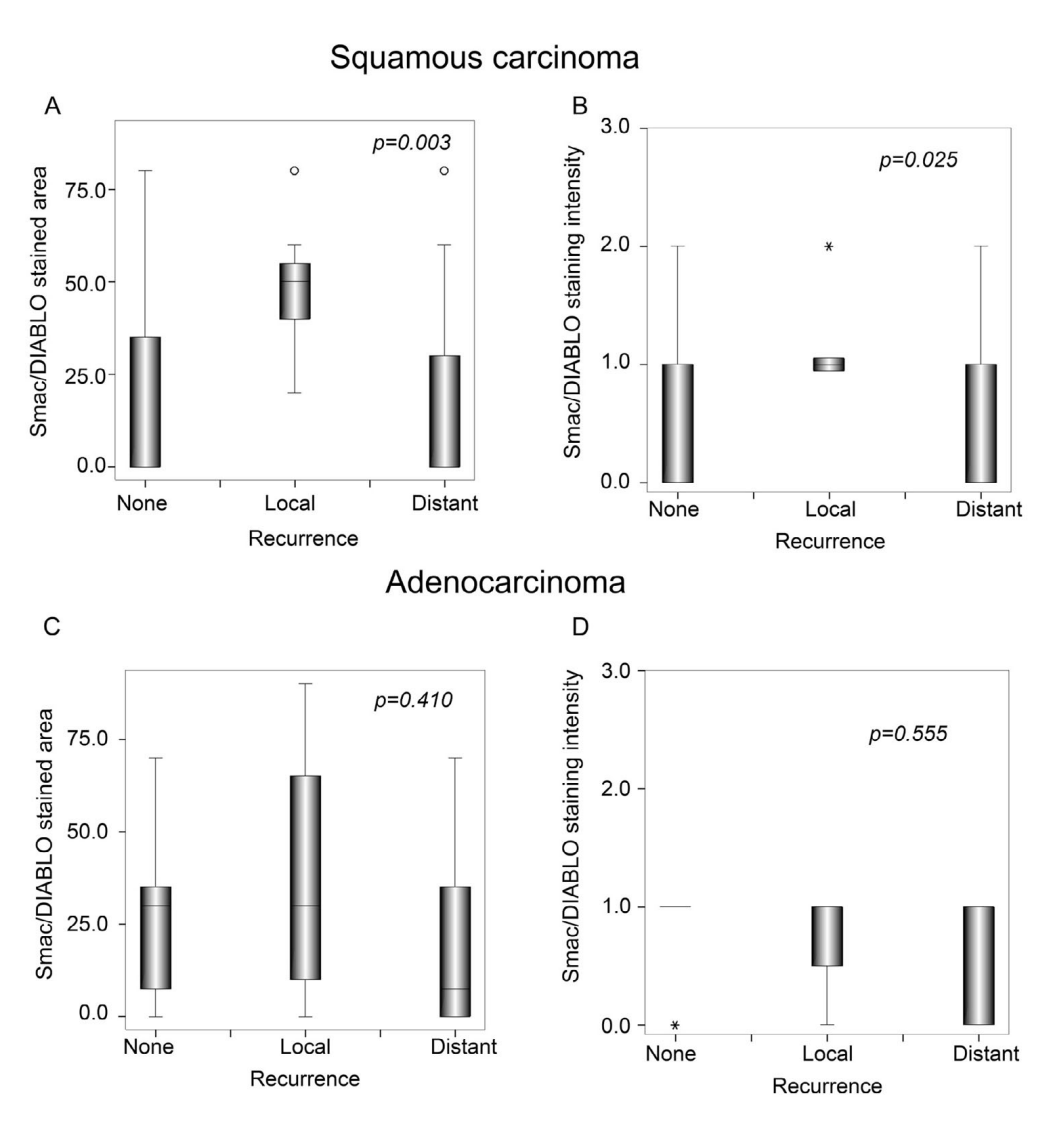
Smac/DIABLO immunostaining correlates with local recurrence in squamous carcinoma. (A) and (C): Smac/DIABLO immunostained area. (B) and (D): Smac/DIABLO intensity of immunostaining. Scores were calculated as described in Material and Methods. Graphs show median, upper and lower quartiles. P value testing the significance of the difference was determined by *Chi *square test.

**Figure 4:**
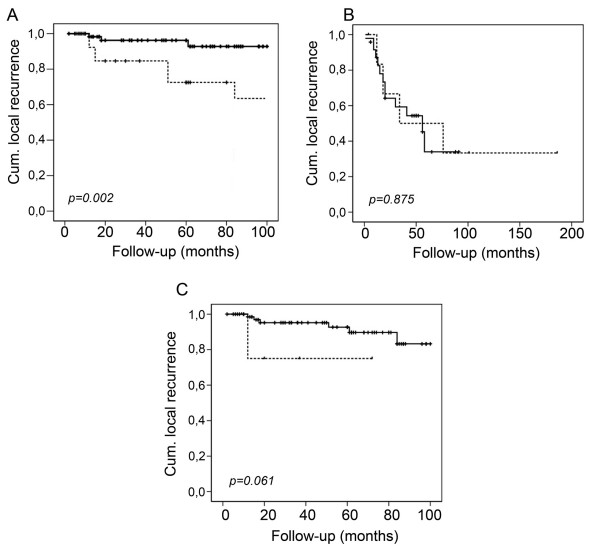
Kaplan-Meier local recurrence estimates in cervical cancer patients by Smac/DIABLO immunostaining. Squamous cell carcinoma samples (A) or adenocarcinoma samples (B), were divided into two groups – low (less than 40 percent) or high (more than 40 percent) immunostained area – and plotted for local recurrence over the time shown (months). Dashed line represents cases with high Smac/DIABLO expression. A similar approach was used for the analysis of intensity of staining in squamous cell carcinoma samples (C). Dashed line represents cases with high intensity. Statistical analysis was performed by log rank test.

**Figure 5:**
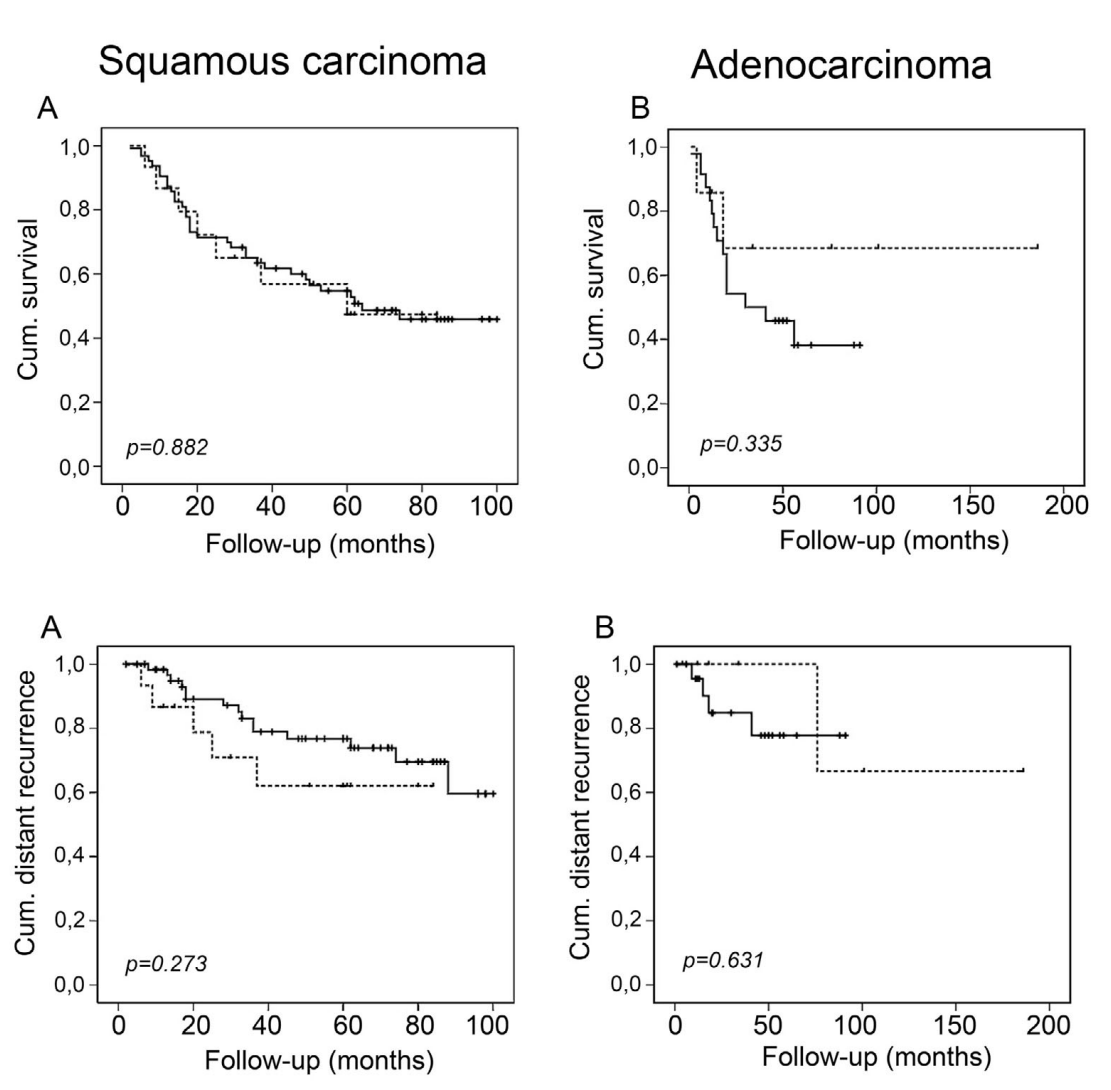
Kaplan-Meier survival estimates in cervical cancer patients by Smac/DIABLO immunoreactive scores. Patients were divided into two groups – low (less than 40) or high (more than 40) scores – and plotted for survival (A and B) or distant recurrence (C and D) over the time shown (months). Dashed line represents cases with high Smac/DIABLO expression. Statistical analysis was performed by log rank test.

To assess whether the level of Smac/DIABLO expression correlated with the clinical variables analyzed, we produced semi quantitative scores of the immunoreactive area and intensity (see Material and Methods). When these scores were analyzed, we found no differences in clinical stage, histology or grade in neither squamous nor adenocarcinoma samples (Table 3 and 4). Similarly, patients with different disease status presented similar scores (Fig. 2). When a more detailed analysis was performed, we found that squamous cell carcinoma patients with local recurrence had higher Smac/DIABLO immunoreactivity than non-recurrent or patients with distant recurrences (p = 0.003) (Fig. 3) No correlation was found for adenocarcinoma samples. To determine whether a higher Smac/DIABLO score could predict local recurrence, we divided the patients into two groups: low (less than 40 percent) or high (more than 40 percent) immunostained area. Patients with squamous cancer in the high score group recurred earlier than those in the low score group (p = 0.002, log rank test) (Fig. 4a). No differences were found for adenocarcinoma samples (Fig. 4b). Interestingly, Smac/DIABLO staining intensity did not correlate with recurrence (Fig. 4c). In addition, both high and low immunostained area groups presented similar survival rates and distant recurrence rates (Fig. 5).

**Table 3:** Smac/DIABLO immunostained area in squamous cancer samples.

Variable	Median Smac score*	*AD*	*p*
**FIGO stage**			
I	-	-	0.55
II	41.25	23.64	
III	33.22	17.59	
			
**Histology of tumors**			
Squamous cell	38.20	21.62	0.36
Adenocarcinoma	34.31	25.41	
			
**Grade**			
Well differentiated	-	-	0.46
Moderately differentiated	35.71	22.43	
Poorly differentiated	39.60	21.50	

* Immunoreactive percentage area of Smac/DIABLO. AD: absolute median deviation. Kruskall-Wallis test was used for Stage and Grade. Mann-Whitney was used for Histology.

**Table 4:** Smac/DIABLO inmmunostained area in adenocarcinoma samples.

Variable	Median Smac score*	*AD*	*p*
**FIGO stage**			
I	54.00	27.01	0.13
II	32.72	24.01	
III	20.83	19.08	
			
**Grade**			
Well differentiated	28.33	22.63	0.56
Moderately differentiated	39.37	219.98	
Poorly differentiated	37	39.97	

*Immunoreactive percentage area of Smac/DIABLO. AD: absolute median deviation. Kruskall-Wallis test was used to test for differences.

## Discussion

Apoptosis is a regulated, energy-dependent form of cell death with a characteristic morphological appearance that includes cellular shrinkage and chromatin condensation. It is central to the carcinogenesis process, allowing tumors to proliferate beyond the constraints that limit normal tissue growth. The importance of alterations in apoptosis during carcinogenesis has been confirmed by the finding that deregulated proliferation alone is not sufficient for tumor formation because there is concomitant induction of cell death [21]. It has been shown that tumors commonly present a marked deficiency in mitochondria-mediated apoptotic pathways. The most studied case is exemplified by the overexpression of anti-apoptotic members of the bcl-2 family in several malignancies [22]. A less explored area concerns the mechanisms that regulate the apoptotic threshold downstream of the mitochondria. In this regard, it has been shown that IAP overexpression is a poor prognostic marker for a variety of solid tumors and hematological malignancies [23]. In addition, IAPs such as survivin are being investigated as diagnostic markers for the presence of occult malignancy [24]. In cervical cancer, we have recently shown that XIAP and survivin are overexpressed and that XIAP levels correlate with relapse of this disease [25].

Smac/DIABLO is a recently-identified proapoptotic protein that interacts with and inhibits several IAPs, including survivin [6,9]. Smac binds to the domain of XIAP that is responsible for binding to processed caspase-9, and thus antagonizes the anti-apoptotic function of XIAP. There is also evidence suggesting that the pro-apoptotic function of Smac/DIABLO could be linked to an additional mechanism, different from IAP binding [26]. The role of this protein during carcinogenesis has not been widely explored. It has been shown that Smac/DIABLO mRNA levels are significantly lower in lung cancers than in normal tissues [16]. Patients with lower Smac/DIABLO mRNA levels have worse prognoses. On the other hand, we recently reported that during cancer progression, a subset of cervical tumors express this protein *de novo *[18]. No clinical correlations were found in that study, perhaps reflecting a specific postraductional modulation of Smac/DIABLO protein. Recent reports have shown that the stability of Smac/DIABLO protein is regulated by its association with several IAPs, including XIAP [27,28], IAP1 and IAP2 [11]. Survivin also binds to Smac/DIABLO, but it is unable to promote ubiquitination. Since both XIAP and survivin are overexpressed in cervical cancer [25], the level of Smac/DIABLO expression should depend not only on mRNA expression, but also on the balance between these proteins and the release from mitochondria. In the present report, we have shown for the first time that Smac/DIABLO protein expression correlates with local recurrence in cervical cancer patients.

The biological reason for this correlation is currently elusive, but several mechanisms could be envisioned. Overexpression of Smac/DIABLO could be related to cancer progression as a response to the enhanced levels of XIAP and survivin, probably selected positively owing to their antiapoptotic activities. This is highly unlikely, since low XIAP mRNA is a marker for earlier recurrence in cervical cancer patients [25]. This decrease could have a similar effect to larger amounts of Smac/DIABLO, as in the present report, so it patients with low XIAP would also be expected to have higher Smac/DIABLO expression. Analyses of both proteins in the same panel of patients should help to clarify this. An alternative explanation for the increased incidence of local recurrence in squamous cell carcinoma patients with high Smac/DIABLO immunoreactivity could be related to the recent finding that Smac binds to NADE, a component of the TRAIL death receptor protein complex [29]. In TRAIL-resistant cancer cells, activation of the receptor for this factor leads to a NF-kappa B-mediated increase in tumor invasion and metastasis instead of cell death [30]. Since direct interaction of Smac/DIABLO with NADE potentiates the signal from the TRAIL receptor, higher Smac expression could lead to an increased invasive potential in resistant cells. Further analysis of the prevalence of TRAIL resistance in cervical tumors should help to clarify this.

An additional contradictory result was the finding of the association of Smac/DIABLO expression with the rate of local recurrence, but not with survival or distant recurrence. Since apoptosis suppression is an important requirement for metastasis, Smac expression could be rendering cervical cancer cells more susceptible to cell death in circulation or, alternatively, to foreign micro ambient compartments, thus counterbalancing the effect of increased local recurrence in prognosis. Supporting this idea, a recent report has shown that XIAP, the main Smac/DIABLO target, is a negative regulator of anoikis of circulating prostate cancer cells [31]. Additional analyses using transgenic cell lines in animal models should help to validate this hypothesis.

Another interesting observation was that the correlation of Smac/DIABLO with local recurrence was restricted to squamous cell carcinoma patients. It has been previously shown that both histological types represent different pathological entities, since adenocarcinoma tumors have poorer prognoses, metastasize more easily to lymph nodes and are more resistant to radiotherapy than squamous cell tumors [32-34]. From these observations, we can expect important biological differences that could potentially explain our data.

One final interesting finding was the association between Smac/DIABLO levels and microvascular density (MVD). Previous reports have shown that MVD is poor prognosis factor in cervical cancer patients [20]. Patients with MVD higher than 20 presented a higher risk of recurrence. Since Smac/DIABLO immunoreactivity was associated with higher MVD, the early recurrence seen in our patients could be due to enhanced angiogenesis. No published reports have explored the effect of Smac/DIABLO overexpression on the synthesis of pro-angiogenic molecules, or vice versa, although the association of Smac with signal transduction complexes [29,35] and the activation of the NF-kappa B pathway could provide a possible mechanism for the synthesis of angiogenic molecules.

## Conclusion

The present report shows that high Smac/DIABLO immunoreactivity is associated with a higher rate of recurrence in cervical squamous cell carcinoma patients. In addition, Smac/DIABLO expression correlated positively with microvascular density in these patients. Further studies directed at elucidating the role of Smac/DIABLO in the recurrence and invasion of cervical cancer are clearly needed.

## Competing interests

The author(s) declare that they have no competing interests.

## Authors' contributions

AAL: Collected the patient data and performed the photomicrography and immunohistochemistry.

FJG: Collaborated in the immunohistochemistry.

DP: Performed the pathology analyses and MVD assays.

DC: Collaborated in clinical assessment of the patients and results.

ME: Collaborated in the pathology analyses

JGD: Collaborated in data analyses and clinical assessment.

VM: Performed the statistical analyses. She also collaborated in editing the manuscript.

JMZ: Designed the study. Coordinated the group. Edited the manuscript.

All authors read and approved the final manuscript

## Pre-publication history

The pre-publication history for this paper can be accessed here:

http://www.biomedcentral.com/1471-2407/6/256/prepub
